# Thrombotic microangiopathy secondary to recurrent prostate cancer 

**DOI:** 10.5414/CNCS110609

**Published:** 2021-09-10

**Authors:** Joseph Newton, Lauren Floyd, Arvind Ponnusamy, John Anderton

**Affiliations:** 1 Manchester University NHS Foundation Trust, Manchester Royal Infirmary, Manchester, and; 2 Lancashire Teaching Hospitals NHS Foundation Trust, Royal Preston Hospital, Preston, Lancashire, UK

**Keywords:** acute kidney injury, hemolytic uremic syndrome, thrombotic microangiopathy, eculizumab

## Abstract

An 86-year-old man returned to the UK from Spain with symptoms suggestive of gastrointestinal bleeding. He was found to have an acute kidney injury and thrombocytopenia. Further investigations identified the presence of a microangiopathic hemolytic anemia, supporting the diagnosis of a thrombotic microangiopathy. Differentials included atypical hemolytic uremic syndrome and secondary thrombotic microangiopathy. Thrombotic thrombocytopenic purpura (TTP) and STEC (Shiga toxin-producing *E. coli*) hemolytic uremic syndrome were excluded by a normal ADAMTS-13 and negative *E. coli* serology and stool PCR. The patient was treated with blood and platelet transfusions. He received eculizumab and hemodialysis whilst a screen for secondary causes was undertaken. Thrombotic microangiopathy was shown to be secondary to recurrence of prostate cancer, which had been treated 16 years previously. He later recovered his renal function and receives ongoing hormonal treatment for his prostate cancer.

## Introduction 

Thrombotic microangiopathy (TMA) is a complex condition in which endothelial damage occurs as a result of thrombus formation in capillaries and arterioles [1] leading to thrombocytopenia, microangiopathic hemolytic anemia (MAHA), and end organ injury [2, 3]. 

Acute thrombocytopenia when associated with acute kidney injury may be due to TMA [4]. This case illustrates an approach to diagnostic workup and demonstrates the difficulty in identifying underlying causes. 

## Case report 

This 86-year-old man was referred to our renal center with a 4-day history of passing black stools and an acute kidney injury (AKI). He had recently returned from a week in Spain and denied any infectious contacts or any significant changes to his diet or activities. 

He described lethargy, rigors, reduced appetite, and a single episode of vomiting. He had noticed bruising in the absence of trauma and reported reduced urine output but no dysuria or obstructive symptoms. He denied any pain, breathlessness, syncope, or weight loss. 

His past medical history included chronic lung disease, polymyalgia rheumatica, hypertension, and a radical prostatectomy for prostatic carcinoma in 2004. There was no significant renal or hematological family history. Regular medicines included low-dose prednisolone, amlodipine, and atorvastatin. He denied any over-the-counter medications, had a good functional status, was an ex-smoker, and drank 15 units of alcohol weekly. 

On examination, he was apyrexial, sclerae were icteric, and there was noticeable bruising over the upper limbs. He was alert and orientated, with a fine tremor but no uremic flap. Respiratory rate was 20 breaths per minute, and oxygen saturations were 97% on room air. Crepitations were present at the right lung base. Heart rate was 73 beats per minute, and blood pressure was 127/73 mmHg. He was warm and well perfused with no peripheral edema. Heart sounds were normal with no pericardial rub. Abdomen was soft and nontender with no palpable organomegaly, and bowel sounds were present. Rectal examination had demonstrated melena, but no comment was made on prostatic size. 

## Results 

Urine culture showed mixed growth but no predominant organisms. Urinalysis demonstrated leukocyturia and hematuria (white cell count 40 ×10^6^/L, red cell count 120 ×10^6^/L). Urine protein-to-creatinine ratio was 16 mmol/mol. Electrocardiogram revealed normal sinus rhythm with infrequent ventricular ectopics. Admission chest X-ray was normal. 

Blood results on arrival are shown in Table 1. The patient had severe AKI, anemia, and thrombocytopenia. Previous renal function in 2018 was normal, with a baseline estimated glomerular filtration rate (eGFR) of 73 mL/min. Blood film revealed schistocytes consistent with a MAHA. Reticulocytes were within normal limits at 72 ×10^9^/L. Iron saturations, vitamin B12, and folate levels were all normal. Ferritin was elevated at 2,281 µmol/L. Coomb’s test was negative. On the third day of admission, results confirmed normal levels of ADAMTS-13. 

Other investigations performed later in the admission included gastroscopy, showing evidence of duodenitis with esophageal candidiasis, and computed tomography of his chest, abdomen, and pelvis, which showed widespread lymphadenopathy but no focal mass or evidence of obstructive uropathy. Virology including HIV, hepatitis B and C were negative. Serology and stool PCR for *E. coli*, legionella, and pneumococcal urinary studies and Leptospira serology were all negative. 

The clinical picture was of a TMA with severe renal involvement. The differentials were between atypical hemolytic uremic syndrome (HUS) and secondary TMA. Thrombotic thrombocytopenic purpura (TTP) and STEC (Shiga toxin-producing *E. coli*) HUS were excluded by a normal ADAMTS-13 and negative *E. coli* serology and stool PCR. 

An extensive screening panel for secondary TMA was requested, and the prostate-specific antigen (PSA) result came back grossly elevated at 2,800 ng/mL. Later histology from a mediastinal lymph node confirmed metastatic prostate cancer, establishing a final diagnosis of secondary TMA. 

Three days into his admission, despite intravenous fluids being given, he developed features of breathlessness and myoclonus thought to be secondary to uremia. Hemodialysis along with blood and platelet transfusions were initiated. On day 4 of admission, he received a dose of eculizumab given the suspicion of a complement-mediated HUS, following meningococcal vaccination and prophylactic antibiotic initiation. He started hormonal treatment for his prostate cancer with a gonadotropin-releasing hormone agonist whilst an inpatient. Only 2 sessions of hemodialysis were required before his renal function recovered. Plasma exchange treatment was not given, on the basis of specialist advice and following rapid access to a negative ADAMTS-13 result. 

He was followed up by the nephrologists and urologists as an outpatient. At 5 months, he was discharged from the renal clinic with an eGFR of 57 mL/min. His complement genetics had revealed no abnormalities. He received ongoing treatment for his prostate cancer in the form of hormonal treatment, and his PSA continues to fall. 

## Discussion 

TMA is associated with a spectrum of diseases, the most common being hemolytic uremic syndrome (further divided into STEC-HUS, and aHUS), and thrombotic thrombocytopenic purpura (TTP). TMA can be subdivided in many ways, one of which is to determine if it is a hereditary TMA or an acquired TMA (Table 2). 

aHUS is a diagnosis of exclusion, where TTP, STEC, and other secondary causes of TMAs have been ruled out [4]. Some of the challenges of definitions have been discussed by Goodship et al. [5], since not all cases of primary (or complement-mediated) aHUS will have a proven complement mutation. A wide range of secondary causes of TMA exist, such as drug-, autoimmune-, and complement-mediated TMA in addition to pre-eclampsia in pregnancy [3, 4]. The possibility of quinine-induced TMA secondary to several gin and tonics that he enjoyed on holiday was considered in our case until further evidence suggestive of malignancy became apparent. Due to the vast spectrum of associated diseases, rarer causes are seldom discussed in case reports. Malignancies including breast, lung, and genitourinary cancers have all been associated with MAHA and secondary TMA [6]. 

There are no specific diagnostic criteria for TMA. The diagnosis is based on evidence of microvascular thrombus, red blood cell destruction, and platelet consumption often evidenced by schistocytes and fragments on a blood film. Other laboratory investigations, such as lactate dehydrogenase (LDH), complements and Coombs test, can be performed to support the diagnosis. Following a diagnosis, the most urgent investigation is to measure ADAMTS-13 (a disintegrin and metalloproteinase with a thrombospondin type 1 motif, member 13) activity in order to diagnose or exclude TTP. In addition, specialist genetic tests can also be performed to determine if there is a genetic or hereditary cause consistent with a complement-mediated HUS (Table 2). 

Treatment options are largely dependent on the underlying cause. Plasma exchange remains a licensed treatment for TTP by removing circulating ADAMTS13 auto-antibodies [4]. The role of corticosteroids remains unclear, and removal of the underlying trigger is often required. 

Complement-inhibiting therapy has now become the mainstay of treatment for TMA secondary to atypical HUS (aHUS). Eculizumab is a monoclonal antibody that binds to the human C5 complement protein and blocks the generation of proinflammatory C5a and C5b-9 [7]. Eculizumab has been used to target the dysregulated complement activity that is inherent to aHUS, often due to underlying abnormal complement genetics (Table 1) [8]. Whilst eculizumab is licensed for use in aHUS, case reports identifying a beneficial use in STEC-HUS, drug-induced aHUS, and malignancy-associated aHUS suggest the possibility of a broader realm of conditions that may benefit [9, 10]. 

**Figure 1.**
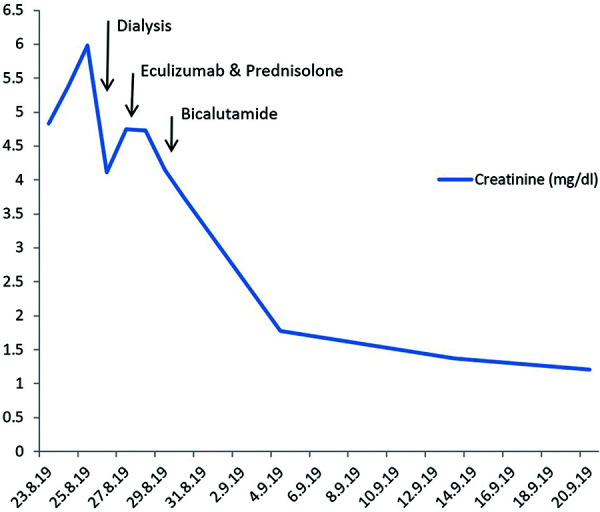
Trend of creatinine and associated interventions at time interval during the admission.

Attributing clinical recovery in our patient to a single dose of eculizumab is debatable. The patient had a marked improvement in his clinical state and biochemical markers including his renal function as shown in Figure 1. Case reports have suggested that eculizumab in secondary aHUS can cause rapid improvement in renal function [10]. In a case series of 29 patients with secondary aHUS, there was marked serological and clinical improvement following treatment with eculizumab. Unlike in our case, the time from diagnosis to treatment was longer at 13 (7 – 26) days, and patients received an average of 6 (3 – 11) doses [10]. Whilst many of the patients treated with eculizumab in the case series by Cavero et al. [10] improved, 6 (20.7%) had no significant improvement in renal function despite evidence of hematological improvement. These findings raise questions regarding the extent of the influence of eculizumab on recovery and whether this may have been in partly attributable to renal replacement therapy or corticosteroids. 

In the case of malignancy-associated TMA, treatment is both supportive and dependent on treatment of the underlying malignancy [11]. Multiple pathways have been identified by which different malignancies may lead to activation of the complement system such as upregulation of complement genes and increased thrombin generation leading to cleavage of C5 [12, 13]. This latter pathway is of interest as this is inhibited by eculizumab, illustrating a potential therapeutic mechanism. It is unclear the extent to which eculizumab supported the recovery of our patient, and we recognize that in many patients with advanced metastatic disease with a poor prognosis, this would not be an appropriate or cost-effective approach. In cases where TMA may have been triggered by certain chemotherapeutic drugs, challenging decisions may have to be taken regarding drug withdrawal [14]. 

A case series from Spain looked at 3 cases of prostate cancer causing aHUS [15]. Similar features were presented in comparison to our patient, with late presentations of advanced prostatic malignancy. Weight loss, dyspnea, bony pain, and clotting abnormalities suggesting disseminated intravascular coagulopathy (DIC) are common features in malignancy-associated TMA [11]. In that case series, a diagnosis of aHUS was made on clinical features. Two patients were treated with plasmapheresis, and none received eculizumab. 

In conclusion, thrombotic microangiopathy should be considered within the differential diagnoses for acute kidney injury presenting with associated thrombocytopenia and evidence of hemolysis. TMA is a complex condition for which many underlying causes must be considered. A new diagnosis of TMA in older patients with a previous history of malignancy should provoke evaluation for progressive malignant disease, and screening investigations should be carried out, even if considerable time has elapsed since curative treatment. Treatment of TMA is often dependent on the underlying cause. However, there may be a role for complement inhibitors, such as eculizumab, in managing patients with non-TTP TMAs with severe renal involvement who are undergoing investigations to identify etiology. 

## Funding 

None. 

## Conflict of interest 

We have no conflict of interest to declare. 

**Table 1.** Biochemistry and hematology results from Day 1 to Day 12 of admission.

	Day 1	Day 3	Day 5	Day 12	
Sodium	132	136	139	142	mEq/L
Potassium	4.1	3.9	4.5	4.4	mEq/L
Blood urea nitrogen	**174.23**	**195.24**	**121.29**	**32.21**	mg/dL
Creatinine	**4.83**	**5.98**	**4.73**	**1.78**	mg/dL
Bilirubin	**5.20**	**1.87**	**1.23**	0.47	mg/dL
Alanine amino-transferase (ALT)	17	27	37	23	units/L
Alkaline phosphatase (ALP)	**210**	**153**	**156**	**167**	units/L
Albumin	3.3	**2.9**	3.4	3.6	g/dL
Adjusted calcium	9.50	9.26	9.10	9.58	mg/dL
Phosphorus	**4.71**	**5.26**	**5.39**	2.85	mg/dL
Hemoglobin	**7.1**	**5.5**	**9.1**	**10.1**	g/dL
White cell count	8,500	10,600	**12,500**	7,800	×10^3^/µL
Platelets	**12,000**	**44,000**	203,000	395,000	×10^3^/µL
International normalized ratio (INR)	1.0			1.0	–
Lactate dehydrogenase (LDH)	**1,431**		**646**		U/L
Results of genetic screening tests					
Genetic screening tests	CFH	0.66 g/L	nmd	CH100	Normal
	CFI	36 mg/Ll	nmd	ADAMTS13	68.5 IU/dL
	Complement C3	1.22 g/L	nmd	ADAMTS-13 inhibitor	Negative
	Complement C4	0.22 g/L	nmd		

nmd = no pathogenetic variant detected. Bold = outside normal reference intervals.

**Table 2.** Hereditary and acquired TMA with the associated diagnostic tests [16, 17, 18].

	Diagnostic tests
Hereditary TMA	
TTP	ADAMTS-13 ADAMTS-13 Inhibitor
Complement-mediated HUS (whilst inherited they are often triggered by causes such as infection or drugs)	Complement studies – C3, C4, complement factor H and I, CD46 expression on neutrophils, CH100, AP100
Metabolism-mediated TMA	Variants in *MMACHC* gene
Acquired TMA	
Pre-eclampsia and HELLP syndrome	Urine dip, Blood pressure, Liver enzymes
Infection related Shiga-toxin, *Streptococcus pneumoniae*, HIV, Epstein-Barr virus (EBV), BK virus	*E. coli* serology and stool PCR Infection screens – pneumococcal and legionella antigen, HIV, EBV and BK serology
Disseminated intravascular coagulation (DIC)	Fibrinogen, clotting factors
Malignancy TMA	Tumor markers, CT imaging, screening tests – mammogram
Drug induced TMA Quinine, interferon, cocaine	
Malignant hypertension	Blood pressure, secondary hypertensive screen – urine catecholamines
